# Progress in Medicine

**Published:** 1901-08-31

**Authors:** 


### Progress in Medicine.

**The Pathology of Dropsy** was discussed at a recent meeting of the Pathological Society. Starling,[1] in opening the subject, said he would limit his remarks to the mechanical form of dropsy due to simple obstruction as met with in uncompensated heart disease. The possible factors were numerous, but in all probability the factor of diminished absorption was negligible compared with those causing increased transudation. The capillary walls presented no opposition to diffusion, and there was always a change going on between the fluid within and that without the walls. The physical factors influenced filtration through the capillary walls, and this depended on (1) the intra-capillary pressure; and (2) the permeability of the vessel walls. The latter varied in different parts of the body—*e.g.* it was much greater in the vessels of the viscera than in those of the limbs. In dropsy, however, the fluid collected in the latter where permeability was most difficult. Rise of pressure alone would not produce œdema; it was necessary to alter the nutrition of the capillary wall—*e.g.* by injection of tissue fibrinogen. Thus everything seemed to depend on the state of the capillary walls: it was certainly so as regards mechanical œdema by pressure of a tumour. But cardiac dropsy was a little different, for apparently there was an actual fall of pressure in the capillaries in simple cardiac failure. It followed, then, that the prime change was not mechanical but in the capillary walls which became damaged by deficient nutrition and were then not able to stand even the normal blood pressure. Lazarus Barlow said he had found, experimentally, that it was possible to increase the venous, and therefore the capillary, pressure very considerably without the slightest evidence that fluid left the blood vessels or passed into the tissues. On the other hand it was easily possible to produce a dropsical condition of the dog's hind paw by completely arresting the flow of blood through the limb for an hour and then allowing it to again flow through the limb. The production of dropsy in this way was brought about more easily if the blood contained in the limb were simply arrested than if the limb were rendered completely anæmic for an hour. This he explained by the supposition that starvation of the limb was a less potent factor than starvation combined with poisoning by the waste products of tissue metabolism. He believed that the real causes of dropsy were to be found in these two factors—starvation of the tissues and their poisoning by waste products. Upon this view the tissues would determine the occurrence of dropsy primarily and not the blood vessels. Sherrington remarked that it might be impossible to separate the action of the vessel wall from that of all the other tissues round it, but the vessel wall was the one constant element and justified the inference that this was the chief seat of change. Herringham said that though the mechanical was not a satisfactory explanation, whatever the immediate cause of dropsy might be, yet it was a factor which could not be ignored; for instance even in generalised renal dropsy if a patient lay on his right side the right side of the face would be more swollen than the left.

**Arterial Hypertonus and Arterio-Sclerosis.**—Russell,[2] in a valuable paper on these conditions, says that the term arterio-sclerosis has been applied by various authors to three conditions:—(1) To atheroma; (2) to a generalised endarteritis; and (3) to a thickening of the intima compensatory to dilatation of vessels from weakening of their middle coat. Russell has examined the arterial system in many cases, and arrived at the following conclusions:—Atheroma and arterio-sclerosis are two totally distinct clinical pathological and clinical entities; whereas atheroma is a localised and patchy affection of the arteries characterised by degenerative changes which have long been recognised; arterio-sclerosis is a generalised affection of the arteries characterised by (*a*) thickening of the tunica media, this thickening being a true hypertrophy, though it may ultimately show some degeneration; (*b*) thickening of the tunica intima from fibrous hyperplasia of the subendothetial connective tissue without atheromatous degeneration; and (*c*) in some cases fibrous thickening of the tunica adventitia. The changes in the arteries in the kidneys differ from those in the radial, or even in the renal arteries themselves before they enter the kidneys, in the following respects: in the kidney the thickening of the intima is proportionately greater, the media is not appreciably thickened, it may even be atrophied. The lumen of the radial arteries and of the arteries in the kidneys is markedly diminished. The changes in the nutrient arteries of the brain, and probably in the cord correspond with those in the arteries inside the kidneys. Arterio-sclerosis may be associated with more or less atheroma in the same subject. Thus Russell's cases contradict Thoma's view that the thickening of the intima is compensatory to weakening and yielding of the media; further, it may in the arteries in the kidneys go on until complete occlusion of the vessels is affected, and it therefore cannot be compensatory. Much of the confusion has arisen from failure to separate the points of similarity and dissimilarity in the changes which occur in the arteries inside and outside the kidneys. Inside the kidney the changes are most marked in the intima, outside the kidney they are most marked in the media. In both, however, the intima is thickened. Inside the kidney the media may disappear and it often atrophies; outside the kidney this never occurs. The clinical diagnosis rests primarily upon the condition of the radial arteries, supplemented by observations on the temporal and other vessels. The uniformity of the thickening distinguishes the condition from atheroma. The arteries have normally a varying amount of tone—*i.e.* the vessel wall is more or less contracted. In disease the muscular coat may be relaxed, as, for instance, in pneumonia, or contracted as in angina and some renal diseases. Hypertonus may be defined as a condition in which the normal tone of the muscular arterial coat is increased; it causes a diminution in the diameter of the blood-vessels. This condition of hypertonus is readily recognised by the educated finger, and such observations can be checked by the use of Oliver's arteriometer. There are also differences in the sphygmographic tracings between a hypertonic and normal condition of the artery. In the former the swing of the lever is less, the percussion stroke is shorter and less abrupt, the summit is rounded and the predicrotic notch less evident. Hypertonus may occur in normal or in sclerosed arteries, and at all ages. In strong people it is associated with, and part of, heightened blood-pressure and a pulse of high tension. In aged people with a failing heart it does not lead to any increase in blood-pressure. The condition is, in the great majority of cases, due to the introduction of poisons into the body or by auto-intoxication. Autointoxication may be caused by physiological errors in digestion, or to errors of katabolism, as in gout, rheumatism, sedentary occupations. The relationship between hypertonus and arterio-sclerosis is as follows:—Recurring or continued hypertonus leads to hypertrophy of the muscular coat of the arteries. The thickened intima is to be explained by the circulation in the blood of deleterious substances of various kinds, which act on the subendothelial connective tissue and lead to its hyperplasia. The evolution of the changes occupies a varying time in different persons; in some hypertonus comes early in life, and in early middle life hypertonus is established, in other sclerosis may not occur until old age.

[1] Starling, Lancet, April 6, 1901. [2] Russell, Lancet, June 1, 1901.

